# Aortic Coarctation Associated With Hypertrophic Cardiomyopathy in a Woman With Hypertension and Syncope: A Case Report With 8-Year Follow-Up

**DOI:** 10.3389/fcvm.2021.818884

**Published:** 2022-01-25

**Authors:** Hong Yang, Hong Wang, Zongzhe Li, Jiangtao Yan, Yu-E Song, Hesong Zeng, Xingwei He, Rui Li, Dao Wen Wang

**Affiliations:** Division of Cardiology and Department of Internal Medicine, Tongji Hospital, Tongji Medical College of Huazhong University of Science and Technology, Wuhan, China

**Keywords:** aortic aoarctation, hypertrophic cardiomyopathy, hypertension, genetic variant, stent repair

## Abstract

**Background:**

Coarctation of the aorta (CoA) is a common congenital cardiovascular malformation with aortic narrowing in the region of the ligamentum arteriosum. Hypertrophic cardiomyopathy (HCM) is a primary cardiomyopathy that is characterized by left ventricular wall thickening and likely left ventricular outflow tract (LVOT) obstruction. They are two irrelevant diseases, and their coexistence has not been reported before. Here, we described a young female patient who concurrently has CoA and HCM.

**Case Presentation:**

The patient has had hypertension since 18-years old and complained of chest discomfort on effort and fatigue thereafter. Initially, she was diagnosed as having hypertrophic cardiomyopathy and primary hypertension. The presence of CoA was not found until she was 35 years old when she had an onset of paroxysmal supraventricular tachycardia (PSVT) and presented with syncope. Failure of the ablation procedure *via* the femoral artery revealed the possibility of CoA and PDA that was confirmed by aortic CTA and angiography. CoA was then treated successfully with a covered stent, and the symptoms of the patient improved remarkably. Additionally, the patient had typical imaging features of HCM, and two novel HCM-causing heterozygous mutations were identified by genetic testing, DSP-encoding desmoplakin, and MYBPC3-encoding myosin-binding protein C. The HCM was suspected to be contributing to the clinical presentations of the patient and challenged the timely diagnosis of CoA. The 8-year follow-up on aortic CTA and angiography revealed no stent graft-related complications. Moreover, no changes in HCM-related imaging features were found in the follow-up echocardiography 8 years after the correction of aortic coarctation, which strengthened the diagnosis of HCM.

**Conclusion:**

Here, we reported the diagnostic challenges, management, and 8-yeasr follow-up findings in a rare case of CoA combined with HCM. The case highlighted the importance for physicians to exclude CoA in young hypertensive patients, and proved the efficacy of stent repair in treating CoA in older patients.

## Introduction

Aortic aoarctation (CoA) represents a spectrum of aortic narrowing that varies from discrete narrowing of thoracic aorta to tubular hypoplasia. Hypertrophic cardiomyopathy (HCM) is defined by the presence of increased wall thickness (usually ≥ 15 mm) in one or more left ventricular myocardial segments and is considered primary cardiomyopathy after exclusion of other cardiac conditions or systemic diseases. In up to 60% of cases, HCM is an autosomal-dominant genetic trait caused by variants of cardiac sarcomere protein genes (1). CoA is a common congenital heart disease, and its prognosis is poor if untreated (2). Clinically, these two anomalies are irrelevant and rarely coexist.

Here, we described the diagnostic challenges, clinical course, imaging studies, management, and 8-year follow-up in an adult female patient who has concurrent presence of CoA and HCM. Patent ductus arteriosus (PDA) and paroxysmal supraventricular tachycardia (PSVT) were also present, which complicated further the clinical features of the patient. Exome sequencing was performed to investigate genetic involvement in the patient. As a result, two likely novel heterozygous HCM-causing mutations in DSP and MYBPC3 genes were identified.

## Case Presentation

A 35-year-old woman presented to our hospital because of repeated episodes of transitory amaurosis and presyncope during the last 3 months. These episodes were often preceded by a short period of palpitation. She also complained of chest discomfort on effort, and she quitted her work as a warehouse manager because of marked fatigue. She had a 17-year history of hypertension and took long-acting nifedipine everyday. She had a 12-year history of mild chest discomfort, and she had been diagnosed as having hypertrophic cardiomyopathy in a local hospital. She also suffered from systemic lupus erythematosus and has been taking oral glucocorticoid for 7 years. She had no family history of hypertension, cardiovascular diseases, or sudden death. The patient had normal vital signs upon admission. Blood pressure (right upper arm) was 135/75 mmHg. There was a systolic murmur at the left sternal border and in second to third intercostal spaces. No other remarkable abnormalities were found during regular physical examination. EKG showed sinus rhythm with right bundle branch block (RBBB) and T wave inversion in precordial, I, and aVL leads.

**Figure 1:**
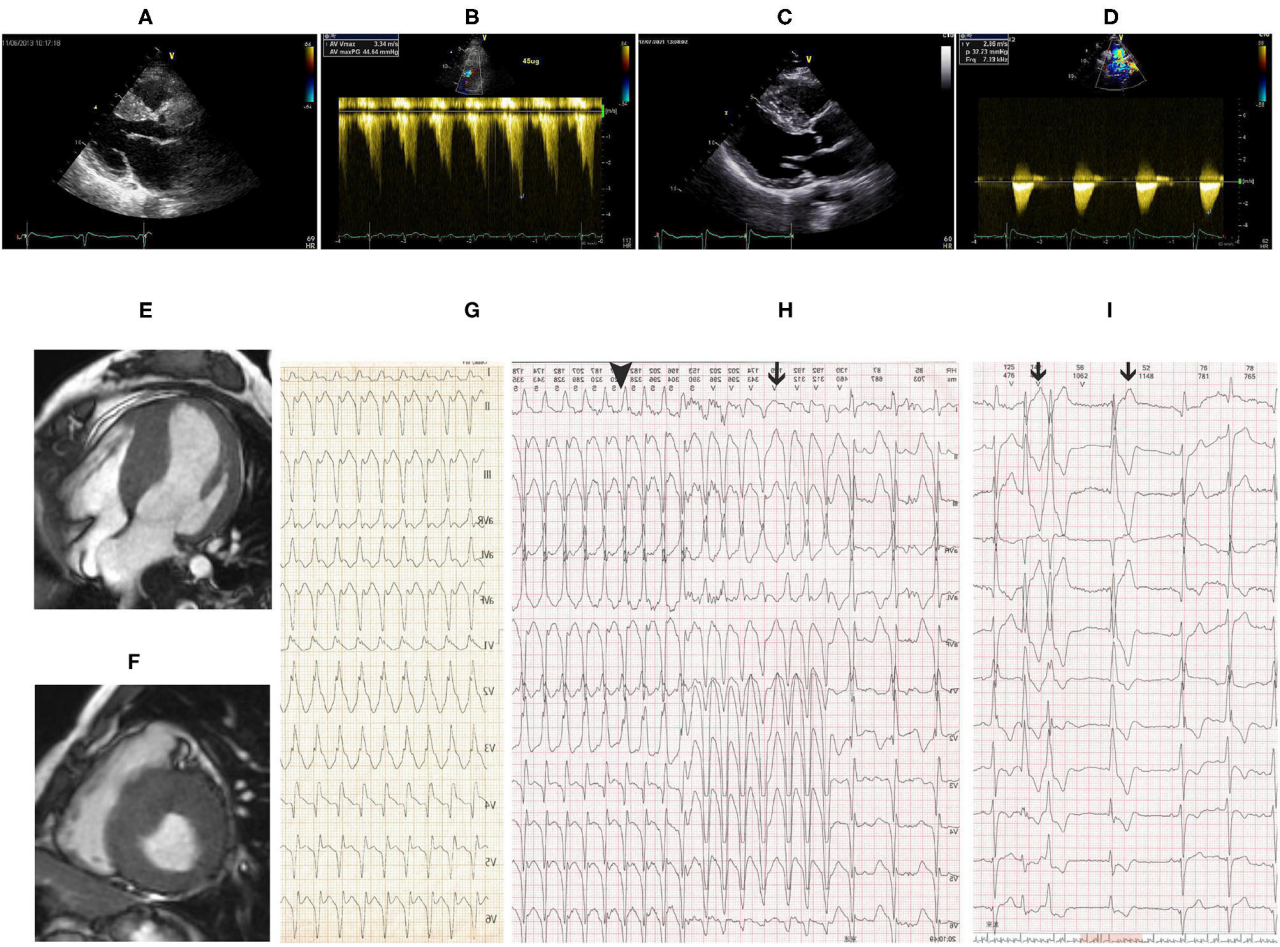
**(A)** Transthoracic echocardiography (TTE) showed remarkably asymmetric left ventricular (LV) hypertrophy with 23-mm thickness of the interventricular septum and 10-mm thickness of the posterior wall. **(B)** Pressure gradient of the left ventricular outflow tract (LVOT) was 44 mmHg after dobutamine infusion at a speed of 45 ug/ml. Follow-up TTE 8 years after CoA correction showed similar LV hypertrophy as before intervention **(C)** and flow velocity at the descending aorta was 2.9 m/s **(D)**. Cardiac magnetic resonance imaging (MRI) showed asymmetric LV hypertrophy in **(E)** apical 4 chamber view and **(F)** short-axis view. **(G)** Twelve-lead echocardiogram (EKG) showed supraventricular tachycardia with right bundle branch block (RBBB) at a heart rate of 190 bpm. Holter monitor **(H,I)** revealed non-sustained ventricular tachycardia (black line in H), supraventricular tachycardia with RBBB (black triangle in H), and premature ventricular beats (black line in I).

Transthoracic echocardiography (TTE) demonstrated asymmetric left ventricular (LV) hypertrophy with abnormal systolic anterior motion (SAM) of the anterior mitral valve leaflet and obstruction of left ventricular outflow tract (LVOT) (Figures 1A,B). There was no critical valvular disease, and systolic function of the left ventricle was normal. Cardiac MRI showed a finding of asymmetric left ventricular hypertrophy as TTE (Figures 1E,F).

On the second day of admission, the patient had an acute episode of palpitation associated with light-headedness. EKG showed supraventricular tachycardia with RBBB (Figure 1G). Electrophysiology study revealed atrioventricular reentrant tachycardia using the left-sided concealed accessory pathway. Catheter ablation procedure was then performed. Surprisingly, *via* the right femoral artery the ablation catheter was failed to access the left heart but the right ventricle, showing left bundle branch block in EKG by stimulation. The ablation procedure was discontinued, and aortic angiography was performed instead. Advancement of the angiographic catheter was blocked at the proximal end of descending aorta, while the wire could be delivered to the right ventricle. Angiography performed on the descending aorta could disclose pulmonary artery rather than aortic arch and ascending aorta.

**Figure 2:**
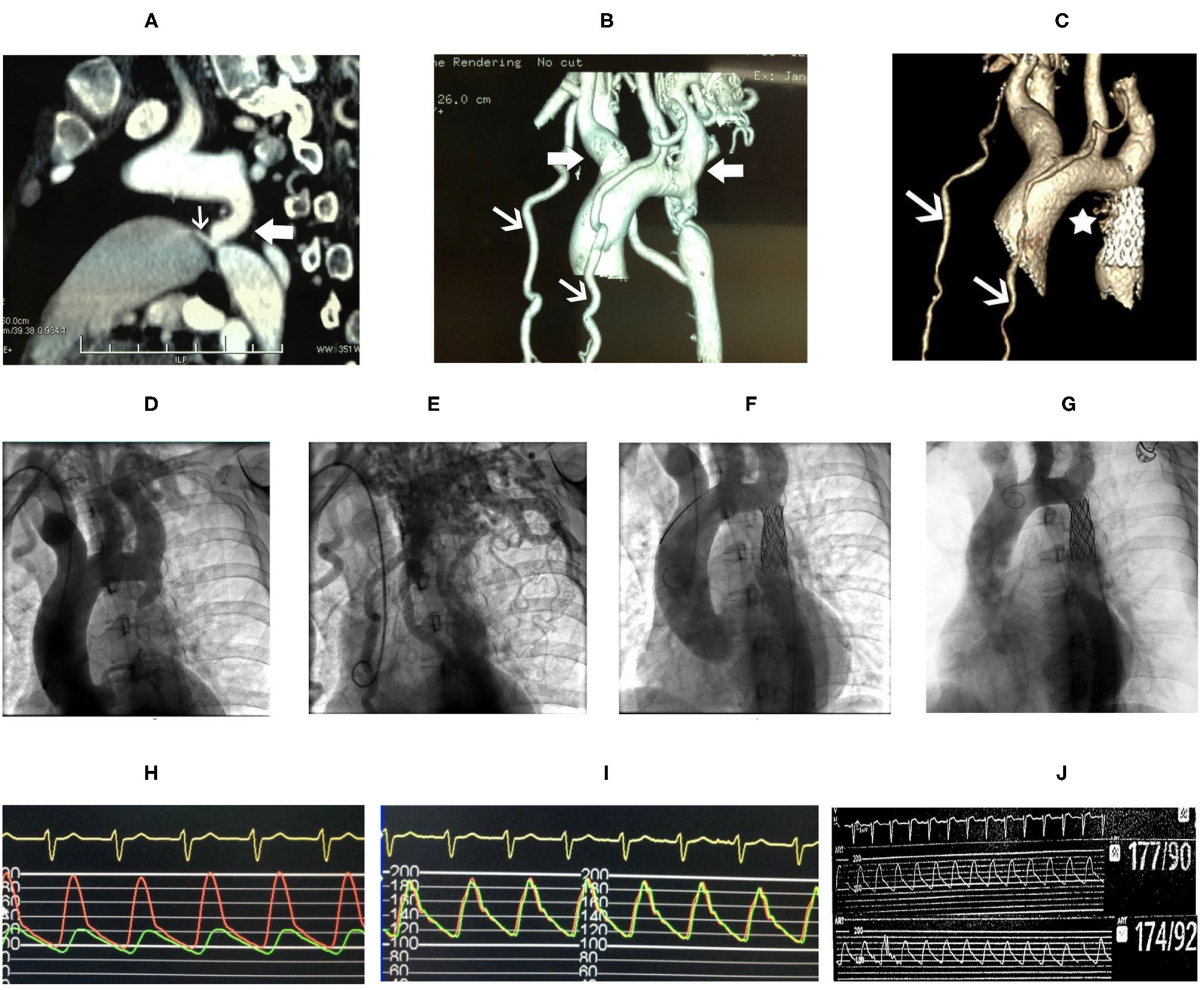
CT angiography (CTA) of the aorta **(A)** showed marked narrowing in the isthmic region of the descending aorta **(white block arrow)** and patent ductus arteriosus (PDA) **(white arrow)** connecting the aorta and the pulmonary artery. Dilatation of the innominate artery and the left subclavian artery **(white block arrows)**, and extensive collateral circulation **(white arrows)** were shown in **(B)**. Follow-up CTA of the aorta 8 years after stent repair showed reduced collateral circulation **(C, white arrows)**, and the PDA is not shown **(C, asterisk)**. Aortic angiography showed remarkable isthmic coarctation of the aorta with dilated innominate artery and left subclavian artery **(D)** and exuberant collateral circulation **(E)**. Pressure gradient between the proximal and distal ends to the aortic obstruction was 74 mmHg **(H)**. The descending aorta, after placement of stent, was shown **(F)** with equalization of pressure at the proximal and distal ends of the descending aorta **(I)**. Repeated aortic angiography at 8-year follow-up showed patency of the covered stent and reduction in collateral circulation **(G)**, and no pressure gradient was found, 177/90 mmHg at the proximal vs. 174/92 mmHg at the distal end of descending aorta, respectively **(J)**.

Coarctation of the aorta (CoA) and PDA were suspected and then confirmed by CT angiography (CTA) of the aorta. The descending aorta was remarkably narrowed at the isthmus by approximately 2 cm past the left subclavian artery (Figures 2A,B). The innominate artery and the left subclavian artery were dilated with extensive collateral circulation arising from those arteries (Figure 2B). A small vessel, immediately distal to the aortic obstruction, was found to be connecting the descending aorta and the pulmonary artery (Figure 2A), confirming the presence of PDA. Blood pressure was measured again, and a pressure gradient of 50 mmHg was noted between the upper and lower extremities. Holter monitor results showed frequent premature ventricular beats (Figure 1I), non-sustained ventricular tachycardia and supraventricular tachycardia (Figure 1H) that had the same morphology as the one captured by 12-lead EKG (Figure 1G).

The catheter ablation procedure was performed again thereafter. An ablation catheter was delivered to the right atrium *via* the right femoral vein and advanced to the left side of the heart by trans-septal puncture. By this way, ablation was completed successfully.

After ablation, the patient had no episodes of presyncope and paroxysmal palpitation. However, she still felt chest discomfort on effort and marked fatigue, and could not go back to work. One month later, she was readmitted to our hospital, and balloon angioplasty with stenting was performed. A catheter was delivered to the root of the aorta *via* the right brachial artery. Angiography of the root of the aorta showed narrowing of the aortic isthmus (Figure 2D) and marked collateral circulation (Figure 2E). Pressure was 200/100 mmHg at the proximal end of the descending aorta to the obstruction and 124/90 mmHg at the distal end to the obstruction (Figure 2H). After balloon dilatation, the covered endovascular stent was placed at the aortic isthmus, and PDA was blocked by the stent concomitantly (Figure 2F). Aortic pressures were equalized at the proximal and distal ends of the descending aorta (Figure 2I). The symptoms of the patient of chest discomfort and fatigue were relieved remarkably after reconstruction. Her blood pressure was below 120/80 mmHg with metoprolol succinate (95 mg/day) that was given to treat premature ventricular beats and ventricular tachycardia and HCM. The patient went back to work one month later after successful repair of CoA. She remained normotensive and was doing well thereafter. On 8-year follow-up, aortic CTA and angiography were repeated, which showed patency of the covered stent (Figures 2C,G), and no pressure gradient was detected at the proximal and distal ends of the descending aorta (Figure 2J). Pressure was 177/90 mmHg at the proximal end and 174/92 mmHg at the distal end of the descending aorta, respectively (Figure 2J). Aortic CTA showed reduced collateral circulation and disappearance of PDA compared to before the stent treatment (Figure 2C). Moreover, repeated TTE on 8-year follow-up showed similar HCM-related imaging features as before the stent treatment, such as similarly thickened interventricular septum (Figure 1C), and flow velocity in the descending aorta was around 2.9 m/s (Figure 1D).

**Figure 3:**
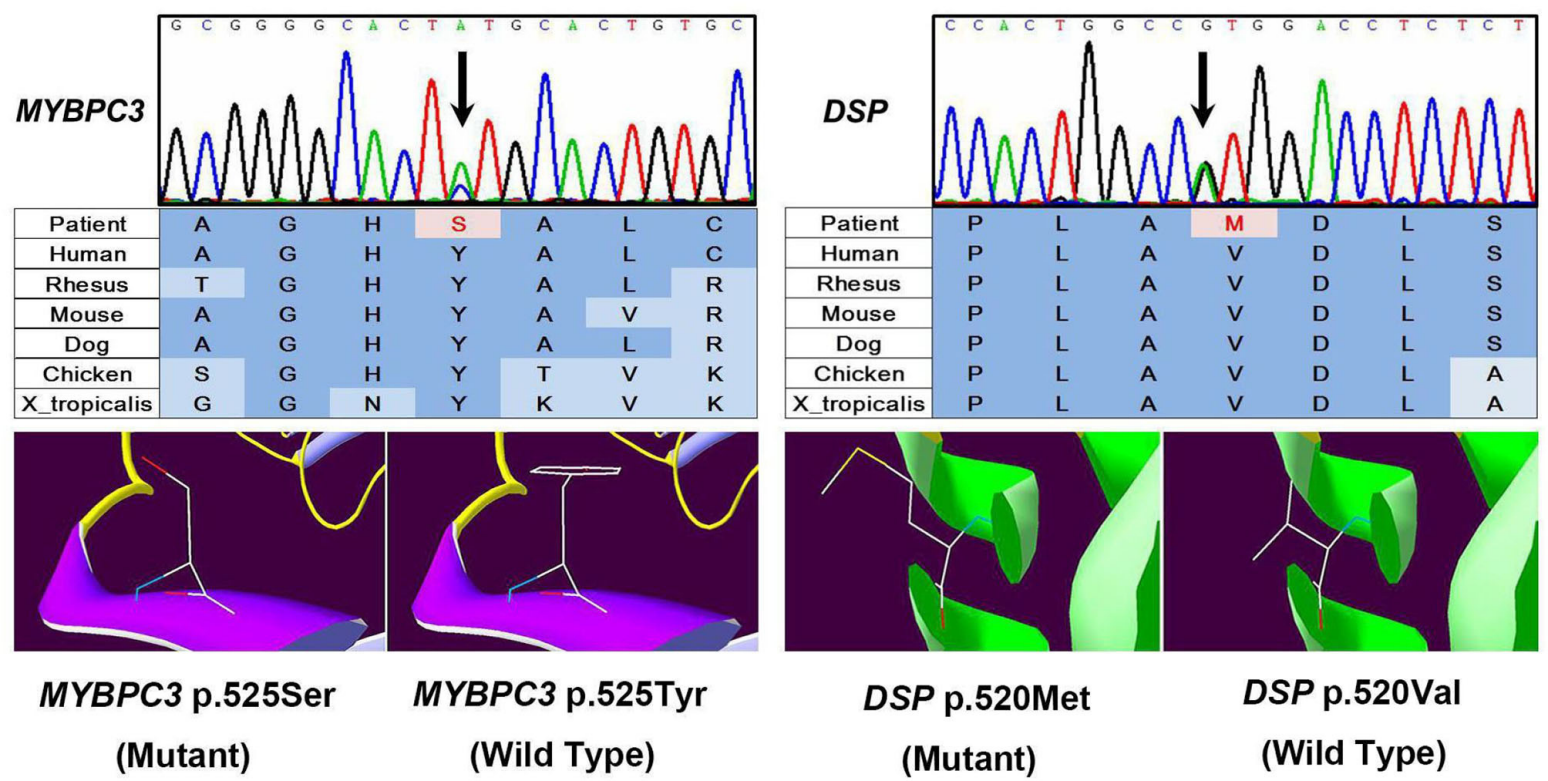
Results of sequencing quality and mutation identification. Two likely novel heterozygous hypertrophic cardiomyopathy (HCM)-causing mutations were identified, DSP (p.Val520Met, c.1558G>A) (SIFT = 0.05; PolyPhen-2 = 1) and MYBPC3 (p.Tyr525Ser, c.1574A>C) (SIFT = 0; PolyPhen-2 = 0.976).

We performed whole exome sequencing with high depth (>100-fold coverage) by next-generation sequencing and then validation by Sanger sequencing to identify genetic involvement in the causation of the disease. After filtering intron variants, synonymous variants, and minor allele frequency >0.005 variants in the dbSNP database, variants in the ESP database, nonpathogenic variants in the ClinVar database, and *in silico* functional predicting, we identified two likely novel heterozygous HCM-causing mutations, DSP (p.Val520Met, c.1558G>A) (SIFT = 0.05; PolyPhen-2 = 1) and MYBPC3 (p.Tyr525Ser, c.1574A>C) (SIFT = 0; PolyPhen-2 = 0.976) (Figure 3) according to the guideline (3). Except for these mutations, we detected no other likely pathogenic mutation in all cardiomyopathy-related genes. Notably, these mutations are located in evolutionary highly conservative areas across multiple species (phylop = 2.6 and 1.94, respectively). Furthermore, the following Sanger sequencing demonstrated that they did not exist in the 500 unrelated ethnically matched healthy controls.

## Discussion

Coarctation of the aorta (CoA) and HCM are two irrelevant cardiac malformations according to anatomy and pathophysiology. These anomalies have not been reported to concurrently exist in the same case. In this case, the clinical features of the patient were complex, and the diagnosis was challenging because of the concurrent presence of CoA and HCM. In addition, PDA and atrioventricular reentrant tachycardia by the accessory pathway were present in the same case, which further complicated the clinical manifestation of the patient. CoA is a common lesion in congenital heart diseases (CHDs), accounting for 6–8% of live births with CHD (4). It can be associated with other congenital defects, such as bicuspid aortic valve, subaortic stenosis, ventricular septal defect, and PDA (5). In this case, CoA was associated with PDA. CoA in adults usually comes to medical attention because of arterial hypertension. The 2008 ACC/AHA guidelines for adults with CHD recommended that every patient with hypertension should have the brachial and femoral pulses palpated to search for “brachial-femoral delay” of CoA, and that blood pressure should be measured in both bilateral arms and legs to search for pressure difference (5). Discrepant blood pressures or pulses between the upper- and lower-extremities are an important sign of CoA. In this case, HCM was diagnosed first by echo during early years of presentation, and the detection of CoA and PDA was delayed until the onset of PSVT. Failure of the ablation procedure for PSVT *via* the femoral artery made the suspicion of CoA and PDA accidently and the following aortic CTA confirmed the diagnosis. Reasons for the delayed diagnosis of CoA in this case include: (1) Presence of HCM complicated the clinical features of the patient, it could explain the patient's presentations as chest discomfort and the murmur on heart auscultation, and made the providers fail to search other possible etiologies; (2) The patient presented with hypertension in 18-years old, but etiology was not fully explored. Blood pressure was only measured in the arms but not in the ankles or legs. Pulse discrepancy between the arms and legs was also ignored; and (3) the initial echo did not include a suprasternal notch view that should reveal high-velocity flow in the descending aorta if done and provide diagnostic clues of CoA.

The prognosis of CoA was poor if untreated, with an average survival age of 35 years and 75% mortality at 46 years of age (2). Therefore, CoA should be diagnosed and treated early in life. Other than treating hypertension, intervention or correction of coarctation should be performed in selected patients. Indications for intervention in adult patients with CoA by the 2008 ACC/AHA guidelines are: (1) peak-to-peak coarctation gradient ≥ 20 mg; which is the difference in peak pressure proximal and beyond the narrowed segment; (2) peak-to-peak coarctation gradient <20 mg with imaging evidence of significant coarctation and radiologic evidence of significant collateral flow (5). So far, there has been no consensus on the choice of surgical repair or percutaneous interventions in older patients. A recent study showed that stent repair of coarctation of the aorta was a safe and feasible alternative to surgical correction (6). For this patient, angioplasty and stenting were performed. Adequate dilatation of the region was achieved, and a covered stent was placed with equalization of pressures in the proximal and distal descending aorta and closure of PDA. After the procedure, the patient was free of major symptoms during the 8-year follow-up and remained normotensive with 95 mg of metoprolol succinate per day. Repeated aortic CTA and angiography after 8 years confirmed persistent patency of the stent graft and revealed no stent-related complications. The clinical and imaging findings of long-term follow-up demonstrated the efficacy of stent repair in treating CoA in older patients.

Hypertrophic cardiomyopathy (HCM) is defined by unexplained cardiac hypertrophy and should be considered only after exclusion of other cardiac or systemic diseases. In this case, increased afterload by CoA and hypertension may also cause LV hypertrophy, and the diagnosis of HCM was questionable. However, in the follow-up, when the CoA of the patient was effectively corrected and her blood pressure was also maintained in the normal range for 8 years, repeated TTE still showed a similar significant LV hypertrophy as before the stent treatment. These follow-up imaging data provide strong evidence supporting the presence of HCM.

Genetic variants in genes that encode different components of cardiac sarcomere protein could explain HCM in up to 60% cases (1). We performed genetic testing in this case and identified a novel mutation in gene-encoding myosin-binding protein C (MYBPC3). MYBPC3 and beta-myosin heavy chain (MYH7) are two large genes accounting for the majority of sarcomere protein gene mutations in HCM (7). Other than MYBPC3, we identified another novel mutation in the DSP gene. DSP makes a protein called desmoplakin that is a crucial component of desmosome structures in cardiac muscle cells. DSP gene mutations have been reported to play a role in arrhythmogenic right ventricular cardiomyopathy, dilated cardiomyopathy, and restrictive cardiomyopathy, but they can present with HCM and atrioventricular block (8, 9). We speculated that mutations in MYBP3 and DSP are contributory to HCM and likely arrhythmias in the patient.

## Conclusion

Here, we reported a rare case of CoA combined with HCM in a 35-year-old woman and discussed the diagnostic challenge, management, and long-term (8 years) follow-up results. The case again highlights that CoA is a rare but important etiology in young hypertensive patients. Moreover, percutaneous interventions such as stent repair are effective treatments of CoA in older patients, as proved by the long-term follow-up aortic CTA and angiography in this case.

## Data Availability Statement

The original contributions presented in the study are included in the article/supplementary material, further inquiries can be directed to the corresponding author.

## Ethics Statement

Written informed consent was obtained from the individual(s) for the publication of any potentially identifiable images or data included in this article.

## Author Contributions

DWW contributed to the conception of the report and critical revision of the report for important intellectual content. HY and HW drafted the manuscript, and were responsible for acquisition and interpretation of the data the searched the literature and revised the figures. ZZL performed genetic testing and analysis. HSZ, JTY, and XWH performed aortic angiography and stenting. Y-ES and RL reconstructed the images and drew the figures. All authors were involved in the treatment of the patient and made a substantial contribution to the preparation of the manuscript, read, and approved the final version of the manuscript.

## Conflict of Interest

The authors declare that the research was conducted in the absence of any commercial or financial relationships that could be construed as a potential conflict of interest.

## Publisher's Note

All claims expressed in this article are solely those of the authors and do not necessarily represent those of their affiliated organizations, or those of the publisher, the editors and the reviewers. Any product that may be evaluated in this article, or claim that may be made by its manufacturer, is not guaranteed or endorsed by the publisher.

## Abbreviations

CoA: coarctation of the aorta
CHD: congenital heart disease
HCM: hypertrophic cardiomyopathy
LV: left ventricle
PDA: patent ductus arteriosus
RBBB: right bundle branch block
TTE: transthoracic echocardiography
LVOT: left ventricular outflow tract.
